# STIM1-dependent Ca^2+^ microdomains are required for myofilament remodeling and signaling in the heart

**DOI:** 10.1038/srep25372

**Published:** 2016-05-06

**Authors:** Cory Parks, Mohammad Afaque Alam, Ryan Sullivan, Salvatore Mancarella

**Affiliations:** 1 Department of Physiology Medicine, University of Tennessee Health Sciences Center, Memphis, TN 38163, USA; 2 Department of Comparative Medicine, University of Tennessee Health Sciences Center, Memphis, TN 38163, USA.

## Abstract

In non-excitable cells stromal interaction molecule 1 (STIM1) is a key element in the generation of Ca^2+^ signals that lead to gene expression, migration and cell proliferation. A growing body of literature suggests that STIM1 plays a key role in the development of pathological cardiac hypertrophy. However, the precise mechanisms involving STIM-dependent Ca^2+^ signaling in the heart are not clearly established. Here, we have investigated the STIM1-associated Ca^2+^ signals in cardiomyocytes and their relevance to pathological cardiac remodeling. We show that mice with inducible, cardiac-restricted, ablation of STIM1 exhibited left ventricular reduced contractility, which was corroborated by impaired single cell contractility. The spatial properties of STIM1-dependent Ca^2+^ signals determine restricted Ca^2+^ microdomains that regulate myofilament remodeling and activate spatially segregated pro-hypertrophic factors. Indeed, mice lacking STIM1 showed less adverse structural remodeling in response to pressure overload-induced cardiac hypertrophy. These results highlight how STIM1-dependent Ca^2+^ microdomains have a major impact on intracellular Ca^2+^ homeostasis, cytoskeletal remodeling and cellular signaling, even when excitation-contraction coupling is present.

The ability of Ca^2+^ to regulate multiple intertwined, yet independent, signaling networks builds upon events that are spatially confined in microdomains and controlled separately. For instance, in cardiomyocytes, cell contraction involves precise control of local Ca^2+^ within the T-tubules, a process known as excitation-contraction coupling (ECC)^1^. However, intracellular Ca^2+^ signaling can lead to activation of target genes responsible for the pathological cardiac hypertrophic response that is often induced by injury to the myocardium or chronic cardiac overload. The source of Ca^2+^ and the spatial distribution of the elements that determine the ECC are well known. Conversely, much debate remains about the source and spatial distribution of the Ca^2+^ pool that activates Ca^2+^-sensitive signaling proteins, such as Ca^2+^-calmodulin–dependent protein kinase (CaMK), calcineurin - nuclear factor of activated T-cells (Cn-NFAT) and the protein kinase C (PKC), each crucial in the conversion to hypertrophy.

Recently, store-operated Ca^2+^ channels (SOCC) have attracted considerable attention as a potential source of Ca^2+^ involved in the activation of transcription factors responsible for the cardiac hypertrophic response^2^,^3^. Stromal interaction molecules (STIM1 and STIM2) are key components of the SOCC, which plays a crucial role in modulating spatial and temporal aspects of Ca^2+^ signaling^4^,^5^. STIM1 is a type 1 transmembrane protein mainly localized in the endoplasmic reticulum, with the N-terminal EF-hand Ca^2+^-binding motif sensing the luminal Ca^2+^ concentration. When intraluminal ER Ca^2+^ concentration falls, STIM1 releases Ca^2+^ from the EF-hand domain oligomerizes and migrates into specialized regions in proximity to the plasma membrane tethering and activating Ca^2+^ selective channels. Ca^2+^ -entry through activated SOCC trigger calcium-activated signaling pathways that couple to downstream gene transcription^6^,^7^,^8^,^9^.

STIM1 expression in the heart shows a dynamic expression profile being up-regulated during fetal development and in the adult heart that is undergoing structural remodeling^3^,^10^, which suggests that its expression is involved in cardiomyocytes growth and remodeling.

STIM1 knockdown *in vitro* is sufficient to prevent phenylephrine-induced neonatal cardiomyocyte hypertrophy^3^,^11^,^12^. Similarly, in the adult rat heart, knockdown of STIM1 is protective against pressure overload-induced hypertrophy^10^. Recently, STIM1 has been reported to bind phospholamban and indirectly activates SERCA2a^13^. On the other hand, STIM1 overexpression in the heart has been linked to higher Ca^2+^ influx associated with increased NFAT and Ca^2+^/calmodulin-dependent kinase II (CaMKII) activity^14^. Moreover, STIM1-deficient mice display impaired cardiac functions that have been linked to morphological and functional mitochondrial abnormalities^15^. However, despite considerable investigation, the underlining mechanisms involving STIM1-dependent signaling in cardiac physiology and pathophysiology remains uncertain.

Current evidence suggests that in non-excitable cells, store operated Ca^2+^ entry (SOCE) couples with the calcineurin-NFAT axis to convey cytoplasmic Ca^2+^ signals to the nucleus where it activates the hypertrophic gene transcription program. However, in cardiomyocytes it is not clear how STIM1-dependent Ca^2+^ signaling co-exists with the more prominent global Ca^2+^ transients that occur with each heartbeat. Indeed, there is a great paucity of information about the precise details of STIM1-dependent signaling and its spatial distribution in cardiomyocytes. So far, it is not clear whether STIM1 forms Ca^2+^ microdomains in cardiomyocytes similarly to those reported for non-excitable-cells after Ca^2+^ store depletion. Importantly, STIM1 has been implicated in the formation of actin stress fibers through RhoA activation^16^, cytoskeletal remodeling through modulation of focal adhesion turnover^17^,^18^,^19^ and in the ER remodeling through its interaction with plus-end-tracking protein EB1 and tubulin^20^. Interestingly, most of these functions are described as independent of SOCE. These properties of STIM1 have been overlooked in the heart.

To decipher the role of STIM1 in cardiomyocytes we conducted a comprehensive analysis of the role of STIM1 in the heart. An in-depth characterization of STIM1-dependent Ca^2+^ signals was conducted using an inducible, cardiac specific, STIM1 knockout mouse combined with *ex-vivo* culture models coupled with live-cell imaging. Our results show that mice with cardiomyocyte-specific STIM1 deletion exhibit a moderate left ventricular dilatation and decreased myocardial contractility. In contrast, STIM1 overexpression reveals formation of subcellular Ca^2+^ microdomains in proximity of STIM1 clusters, which facilitate myofibrillar protein degradation. Finally, we report that acute STIM1 genetic deletion attenuate pressure overload-induced cardiac hypertrophy by preventing focal adhesion activation and its downstream signaling. Our data reveal previously unidentified STIM1-dependent intracellular Ca^2+^ microdomains that are required for normal cardiac homeostasis and fibrillar remodeling during hypertrophy.

## Results

### STIM1 KO mice exhibit decreased cardiac function

To examine the physiological role of STIM1 in the heart we generated an inducible cardiac-specific STIM1 knockout mouse. Tamoxifen inducible α-MHC-MerCreMer transgenic mice (Cre transgene under control of the α-myosin-heavy-chain promoter), were crossed with STIM1^flox/flox^ mice and offspring backcrossed until STIM1^flox/flox^-Cre^+/−^ mice were generated (designated as “mutant” in this study). STIM1^WT/WT^-Cre^+/−^ was the designated control. This mouse model allows tight gene regulation, however, a careful investigation was conducted to establish the dosage of tamoxifen that maximize Cre-recombinase activity and minimize tamoxifen side effects^21^,^22^. The Cre-recombinase temporal and spatial patterns were determined by crossing the α-MHC-MerCreMer mice with mT/mG reporter mice (double-fluorescent reporter mice that express membrane-targeted tandem dimer tomato (tdTomato) before Cre-mediated excision and membrane-targeted GFP (mGFP) after excision^23^,^24^. Immunostaining of hearts harvested from (mT/mG)-Cre^+/−^ mice 7 days after receiving the last injection of tamoxifen (i.p., 40 mg/kg/day), revealed that Cre-recombinase activity peaks after 3 injections (Supplemental Fig. S1a). Since this paradigm has not been associated with tamoxifen-induced cardiotoxicity^25^ and was sufficient to induce ~80% reduction of STIM1 proteins in freshly isolated cardiomyocytes as assessed by Western blot (Supplemental Fig. S1b,c), we used this approach throughout this study.

**Figure 1:**
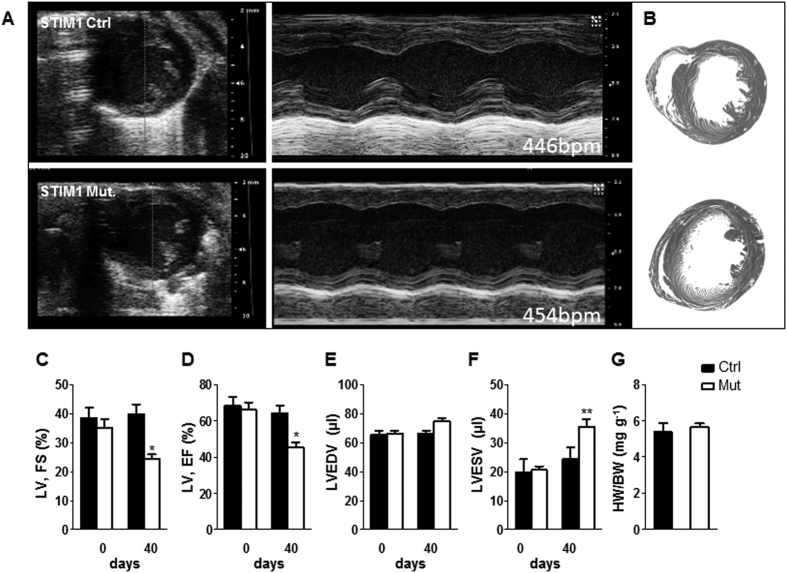
Echocardiography at 0 and 40 days after tamoxifen injection reveals decreased cardiac performances in STIM1 mutant mice. (**A**) Representative ultrasound images of LV (short-axis) in B-mode and M-mode (at the level of the papillary muscles) from STIM1 mutant and control mice. (**B**) Representative heart cross-sections from control mice (upper panel) and STIM1 mutant mice (lower panel). (**C**–**F**) Averaged LV parameters obtained from analysis of echocardiograms: (**C**) fractional shortening (FS); (**D**) ejection fraction (EF); (**E**) left ventricular end-diastolic volume (LV-EDV); (**F**) left ventricular end-systolic volume (LV-ESV). (**G**) Ventricular weight/body weight ratio (mg of left ventricle/g of body weight), data taken 40 days post injection, (control hearts *n* = 6; STIM1 mutant, *n* = 5). Values represent mean ± SEM, echo data were obtained from n = 11 controls and n = 16 STIM1 mutant mice. *P < 0.05, **P < 0.01.

Serial echocardiography was used to determine the functional consequences associated with STIM1 deletion (Fig. 1A). The mutant and control^−^ mice underwent ultrasound interrogation before (day 0) and after (40 days) after tamoxifen treatment. Echocardiographic results were validated postmortem (Fig. 1B). Left ventricular function was similar between control and mutant mice before treatment (Fig. 1C–F). On day 40 mice appeared healthy, however, there was evidence of decreased left ventricular (LV) contractility as indicated by a decline in fractional shortening (39.81 ± 9.36% in controls vs. 25.41 ± 4.65% in the mutant mice, *p* < 0.05), which was associated with reduced ejection fraction (64.33 ± 11.56% from control hearts vs. 45.44 ± 7.9% from mutants; *p* < 0.05) (Fig. 1D). Moreover, STIM1 mutant mice showed a mild dilatation of the left ventricle associated with a moderate increase in end-diastolic volume (Fig. 1E) and a more pronounced increase in end-systolic volume (Fig. 1F). Left ventricular weight to body weight ratio was not statistically different 40 days post-injection, averaging 5.3 ± 0.5 mg/g in mutant mice and 5.6 ± 0.5 mg/g in control mice (Fig. 1G).

### STIM1 deletion results in impaired cell contractility and altered [Ca^2+^]_i_ cycling

Since STIM1 plays a crucial role in regulating [Ca^2+^]_i_, we investigated whether STIM1 deletion altered cardiomyocyte contractility and [Ca^2+^]_i_ cycling in isolated adult cardiomyocytes. Presence of functional SOCE was assessed in fura-2 loaded cells where Ca^2+^ was pharmacologically depleted from the intracellular stores (see methods for details). Upon re-introduction of extracellular Ca^2+^, fura-2 ratio signal increased significantly in wild type cardiomyocytes, compared to the smaller fura-2 signal in cardiomyocytes isolated from STIM1 mutant hearts (Supplemental Fig. 2A,B). This result suggest presence of STIM1-dependent Ca^2+^ entry component in cardiomyocytes.

**Figure 2:**
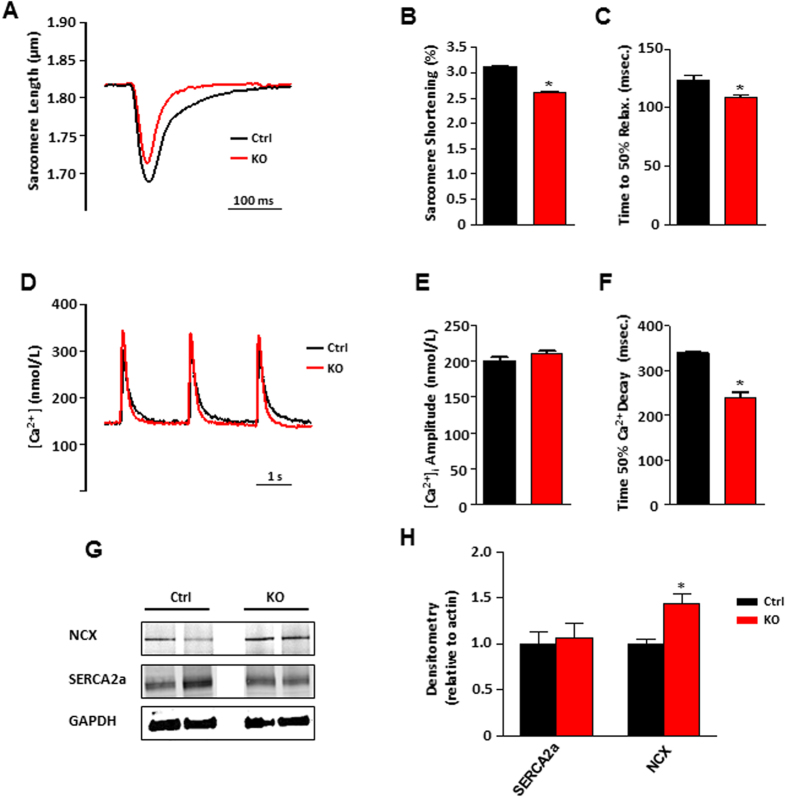
Sarcomere shortening and intracellular Ca^2+^ recordings from STIM1 controls and STIM1 mutant acutely isolated cardiomyocytes. (**A**) Representative superimposed traces showing sarcomere length changes during electric-pulse stimulation (0.5 Hz). (**B**) Averaged data of sarcomere shortening amplitude (percentage of baseline). (**C**) Average data showing time to 50% relaxation (Ctrl, *n* = 26 cells from 5 mice; KO, *n* = 62 cells from 5 mice). (**D**) Representative normalized and superimposed electrically-evoked (0.5 Hz) [Ca^2+^]_i_ transients from STIM1 Ctrl (black trace) and STIM1 KO cardiomyocytes (red trace). (**E**) Averaged peak amplitude of [Ca^2+^]_i_ transients. (**F**) Time to 50% [Ca^2+^]_i_ transient decay, STIM1 KO 240 ± 6 ms, n = 18; vs. the control 340 ± 8 ms, *n* = 24. Cells were isolated from 6 hearts from each group. (**G**) Typical Western blot results for SERCA-2 and Na^+^–Ca^2+^-exchanger. (**H**) Average data quantification of Western blot data for SERCA2a and NCX1, total proteins were extracted from adult isolated cardiomyocytes obtained after whole heart digestion. Cells were extracted from 8 hearts (4 each group). GAPDH was used as loading control, *P < 0.05.

In another set of experiments, single cell contractility and intracellular Ca^2+^ transients were investigated in electrically-paced cells (0.5 Hz). Consistent with the echocardiographic results indicating reduced myocardial shortening, STIM1 KO cardiomyocytes displayed decreased contractility as determined by reduced sarcomere length to peak amplitude and reduced sarcomere shortening (Fig. 2A,B). Furthermore, STIM1 KO cardiomyocytes relaxed faster as indicated by the time to 50% relaxation (Fig. 2C). Fura-2 loaded cardiomyocytes from STIM1 KO and control cells showed similar Ca^2+^ transients peak amplitude (Fig. 2D,E). However, in STIM1 KO cells cytosolic Ca^2+^ clearance rate was enhanced when compared with cardiomyocytes from the control group (Fig. 2F). The SR Ca^2+^ content, assessed by local caffeine application (10 mM) revealed no difference between control and STIM1 KO cells (Supplemental Fig. 2C). We next examined if altered expression of SERCA2a or the sodium/calcium exchanger (NCX) could account for the fast Ca^2+^ clearance observed. Western blot quantification showed that SERCA2a protein level was unchanged, however the Na^+^/Ca^2+^ expression was significantly upregulated (Fig. 2G,H). Taken together, these results suggest that STIM1 deletion results in cytosolic Ca^2+^ homeostasis perturbation and it is associated with defects in contractility of cardiac myocytes.

### Altered vinculin distribution and microtubule network disarray in STIM1-deficient cardiomyocytes

**Figure 3:**
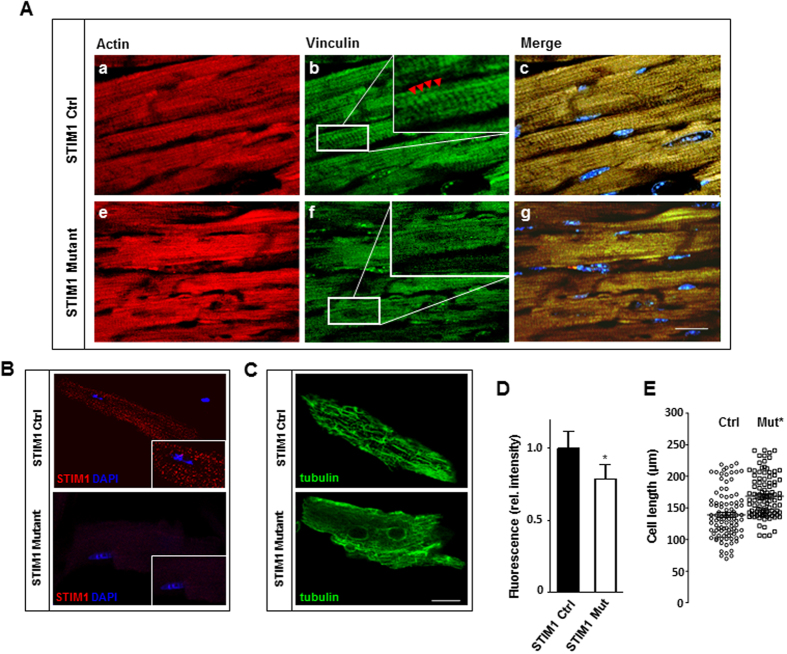
STIM1 genetic deletion results in heterogeneous vinculin distribution and myofibrillar rearrangements. Hearts were harvested 40 days after tamoxifen treatment. (**A**) Representative immunostaining of heart sections. Actin was labeled with rhodamine-phalloidin (red) to identify cardiomyocytes (a–e), vinculin, in green, is detected by anti-vinculin antibody (b–f), nuclei in blue are stained with DAPI (c–g). In control hearts vinculin immunofluorescence staining shows costameres with intensely stained intercalated disks indicated by red arrowhead (panel (b) and inset). Heart sections from STIM1 mutant showed abnormal staining and loss of organized costamere and intercalated disk structures (panel (f) and inset). (**B**) Immunolocalization of endogenous STIM1 (red) and DAPI (blue) in isolated adult mouse cardiomyocytes. (**C**) Immunofluorescent confocal imaging showing the distribution of total β-tubulins (microtubular network), in isolated adult mouse cardiac myocytes. (**D**) Quantitation of β-tubulin, fluorescence intensity was quantified from a minimum of 50 cells per group (4 mice each group). (**E**) Scatter vertical graph of cardiomyocytes cell length, mean and error bars are also reported, STIM1 KO *vs.* Ctrl cells. *P < 0.05. Scale bar: (**A**) = 25 μm and (**B**) = 15 μm.

Reduced fractional shortening and impaired cell contractility suggests that the contractile apparatus could be compromised as a consequence of STIM1 deletion. Interestingly, it has been widely reported that STIM1 is involved in the regulation of myofilaments reorganization, indeed, STIM1 interacts with proteins of the cytoskeleton to maintain actin architecture by assembling/disassembling focal adhesions during cell migration^18^,^20^,^26^. Although cardiomyocytes are differentiated cells that have lost their ability to migrate, focal adhesion complexes persist forming scaffolds of proteins essential for mechanosensing and mechanotransduction pathways in the heart^27^,^28^. Therefore, we hypothesize that STIM1 deletion may cause abnormal function of the elements in the contractile apparatus by affecting focal adhesion sites. To further validate our hypothesis, we analyzed the intracellular distribution of vinculin, a membrane-bound cytoskeletal protein and crucial component of focal adhesion complex. Vinculin is contained within costameres and intercalated disks and plays a key role in mediating contraction force transmission from cardiomyocytes to the extracellular matrix. Immunofluorescence analysis of control left ventricle stained with anti-vinculin antibody displays the characteristic normal pattern of staining with parallel sarcomeres which coincided with that of the T-tubular system (Fig. 3A). In contrast, the left ventricular myocardium of STIM1 mutant showed a poorly distinguishable pattern of costamere staining (Fig. 3A), in these areas, the costameres and the intercalated disks appeared diffuse and of low intensity (Fig. 3A inset).

Immunocytochemistry revealed presence of STIM1 in isolated adult cardiomyocytes with a “cluster-like” arrangement, rather than a uniform SR distribution as typically observed in non-excitable cells (Fig. 3B). No signal was detected by anti-STIM1 in STIM1 KO cells, further validating the specificity of the antibody (Fig. 3B). Cardiomyocytes from control hearts stained for tubulin, exhibited a branched microtubule network, whereas in STIM1 KO cells the distribution of tubulin was diffusely spotted and fragmentary with filaments irregularly distributed (Fig. 3C,D). Moreover, STIM1 KO cells were more elongated as compared with cells from control mice (Fig. 3E). Hence, cytoskeletal remodeling is consecutive to STIM1 deletion and provides further support to the observed LV dilatation. Taken together these results show that STIM1 is critical in the distribution and organization of myofilaments within cardiac myocytes.

### STIM1 overexpression results in myofibrillar disarray, focal adhesion disassembly and defective cell contraction

**Figure 4:**
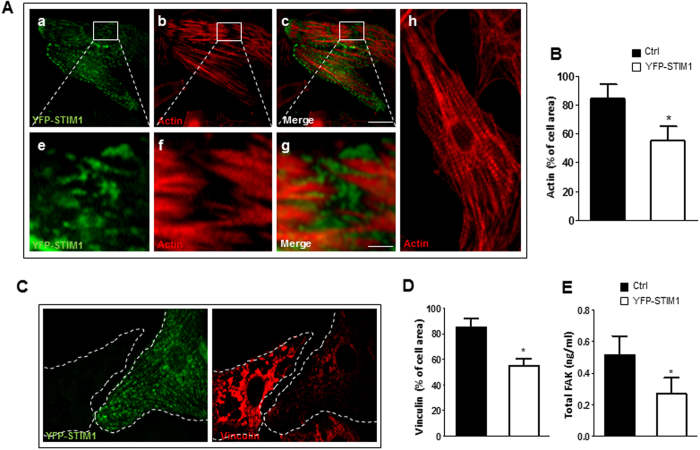
STIM1 overexpression in NVCM leads to myofibrillar remodeling and downregulation of focal adhesion kinase. NVCM were infected with lentiviral YFP-STIM1. (**A**) NVCM were fixed, and identifiable by actin content labeled with rhodamine-phalloidin (a), co-stained with anti-GFP antibody to selectively visualize YFP-STIM1 (b). Bottom panels show representative fields displaying sites where STIM1 expression leads to loss of myofibrillar organization (e–g). This is in striking contrast with the regular actin pattern exhibited from non-infected control cells (h). (**B**) Bar graph showing average values of actin as percentage of total cell area in control cells or cells over-expressing STIM1 respectively (**C**) Representative images of NVCM stained with anti-GFP against YFP-STIM1 (green) and anti-vinculin (red). Dotted lines represent cell edges. (**D**) Histogram representing vinculin areas of control cells or cells expressing STIM1 respectively. Vinculin fluorescence intensity, values are normalized to the mean value of control cells. Values are represented as mean ± SEM (4 fields of view, three independent experiments). (**E**) Total FAK quantification from control and STIM1-YFP overexpressing cardiomyocytes. Intracellular FAK content was obtained with ELISA kit, bar graph representing signal emission at 510 nm. Experiments were assayed in triplicate. Data were plotted after correction for total protein quantification. Values represent mean ± SEM, n = 3 independent experiments *P < 0.05. Scale bar: 50 μm.

In contrast to the developing heart and during pathological cardiac hypertrophy, STIM1 expression in normal adult cardiomyocytes is very low^3^,^10^. Therefore, cultured rat neonatal ventricular cardiomyocytes (NVCM), represent an excellent model to investigate the biological effect normally associated with STIM1 induced expression. Surprisingly, NVCM infected with lentiviral particles transducing for YFP-STIM1 displayed profound alteration in the organization of the actin-cytoskeletal meshwork. In particular, confocal images of NVCM overexpressing YFP-STIM1 stained with rhodopsin conjugated phalloidin clearly displayed STIM1 clusters localized at the actin endpoints affecting focal adhesion structure causing severe myofibrillar degeneration (Fig. 4A,B). Indeed, images obtained with confocal microscopy revealed that STIM1 over-expression dramatically changed vinculin distribution (Fig. 4B–D). Consistent with a derailed myofilament organization, live NVCM expressing YFP STIM1-displayed unbalanced contraction, resembling a wobbling motion rather than a well-coordinated contraction (Supplemental Data S3). Furthermore, cardiomyocytes over expressing STIM1 showed reduced focal adhesion kinase (FAK) expression as compared to non-infected cells (Fig. 4E)

Collectively, these results provide evidence that STIM1 accumulation in subsarcolemmal space form clusters that modulate focal adhesion assembly and disassembly to achieve uniform force transmission.

### STIM1 clusters increase cytosolic resting Ca^2+^ concentration

**Figure 5:**
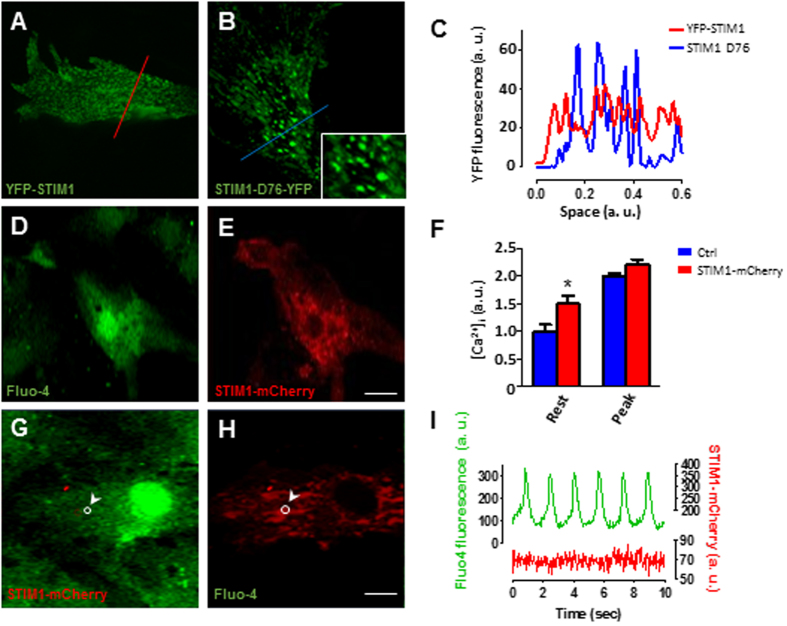
STIM1 overexpression forms puncta-like structures and alters intracellular Ca^2+^ dynamics in NVCM. (**A**) Representative images from live-cell imaging of cultured NVCM infected with lentiviral YFP-STIM1. (**B**) NVCM expressing STIM1-D76-YFP, in the inset it is possible to appreciate STIM1-D76 clusters at higher magnification. (**C**) Intensity profile of YFP-STIM1 fluorescence along the red line from the image in panel (**A**) (red trace); STIM1-D76-YFP fluorescence intensity profile along the blue line from the image in panel (**B**) (blue trace). (**D**) Representative confocal image of a live NVCM loaded with fluo-4 and excited at 488 nm. (**E**) Same cells as in “panel d” excited at 587 nm reveling one cardiomyocyte expressing STIM1-mCherry. (**F**) Intracellular Ca^2+^ concentration levels between control and STIM1-mCherry transfected cells. Measurements were taken taken at baseline and at the peak of the [Ca^2+^]_i_ transient then compared. (**G**,**H**), Cardiomyocyte loaded with fluo-4 and expressing STIM1-mCherry, a region of interest (ROI) is selected (white circle). (**I**) Simultaneous measurements changes of fluorescence intensity within the ROI (panel (g,h)) during [Ca^2+^]_i_ transient (green trace, emission 515 nm) and STIM1-mCherry fluorescence changes (red, emission 610 nm). Scale bar (**A**–**E**) = 10 μm; G,H 20 μm.

To understand how STIM1 is involved in myofilament disarray and focal adhesion composition, we first examined STIM1 distribution in beating NVCM. Confocal live-cell imaging of NVCM expressing STIM1-YFP demonstrated that STIM1 forms spontaneously occurring and clearly distinguishable STIM1 clusters (Fig. 5A), which can be observed in both contracting and non-contracting cardiomyocytes. Since one of the distinctive characteristics of STIM1 is its ability to oligomerize, it was important to establish whether in cardiomyocytes STIM1 can further oligomerize upon Ca^2+^ store depletion. NVCM were infected with the constitutively active YFP-STIM1 (D76A) mutant, in which the first Ca^2+^ binding aspartic acid residue in the EF-hand is substituted with alanine. The STIM1-D76A is insensitive to the Ca^2+^ in the SR lumen mimicking the Ca^2+^ dissociated state of STIM1, hence, STIM1-D76 oligomerize even in absence of store depletion (store full)^19^,^29^,^30^. Interestingly, when STIM1-D76 was expressed in NVCM a strong fluorescence signal was located at the center of the STIM1 clusters suggesting that STIM1 can undergo further aggregation (Fig. 5B,C). Next, we examined the impact of STIM1 expression on [Ca^2+^]_i_ transients. Cells were grown on a glass coverslip and transfected with STIM1-mCherry. After 48–72 hours post transfection cells were loaded with fluo-4 AM and under these conditions it was possible to image [Ca^2+^]_i_ transients from one or two STIM1-mCherry expressing cells per selected field (40×) and compare it to the [Ca^2+^]_i_ transients from non-transfected cells (Fig. 5D,E). Analysis of either spontaneous or electrically-induced [Ca^2+^]_i_ transients revealed that, resting [Ca^2+^]_i_ level was significantly higher in STIM1 over-expressing cells than neighboring non-infected cells, while peak amplitude of the Ca^2+^ transients was little affected (Fig. 5F and Supplemental S4a). No difference was detected between Ca^2+^ transients amplitude or contractility between GFP infected cells and non-infected cells loaded with calcium indicator X-Rhod-1. Furthermore, Ca^2+^ transients analysis revealed that STIM1 overexpression caused a significant prolongation of the time to 50%-decay of [Ca^2+^]_i_ (Supplemental Fig. S4b,c). Next, these findings were validated in adult cardiomyocytes. Short-term cultured adult rat cardiomyocytes overexpressing STIM1-YFP were loaded with X-rhod-1 to detect [Ca^2+^]_i_, changes, indeed, YFP-STIM1 overexpression was associated with a significant increase in [Ca^2+^]_i_ level as compared to cells non expressing STIM1-YFP (Supplemental Fig. 5A,B).

STIM1 is a highly mobile protein that oligomerizes in response to changes in the SR Ca^2+^ content. Indeed, we have shown that despite STIM1 spontaneously clusters in cardiomyocytes, independently from the Ca^2+^ concentration in the SR, further oligomerization is possible as suggested by STIM1-D76 expression in NVCM. Therefore, we investigated whether the drop in SR Ca^2+^ concentration that occurs at each transient correlates with STIM1 intracellular rearrangements. NVCMs expressing STIM1-mCherry loaded with fluo-4 were used to simultaneously visualize STIM1 movements and intracellular Ca^2+^ events (Fig. 5G,H). Region of interest analysis showed that while fluo-4 intensity changed according to intracellular Ca^2+^ fluctuation, STIM1-mCherry fluorescence intensity did not change during the [Ca^2+^]_i_ cycle, suggesting that STIM1 is not involved in the cardiac ECC (Fig. 5I).

### STIM1 induces spatially restricted Ca^2+^ microdomains

**Figure 6:**
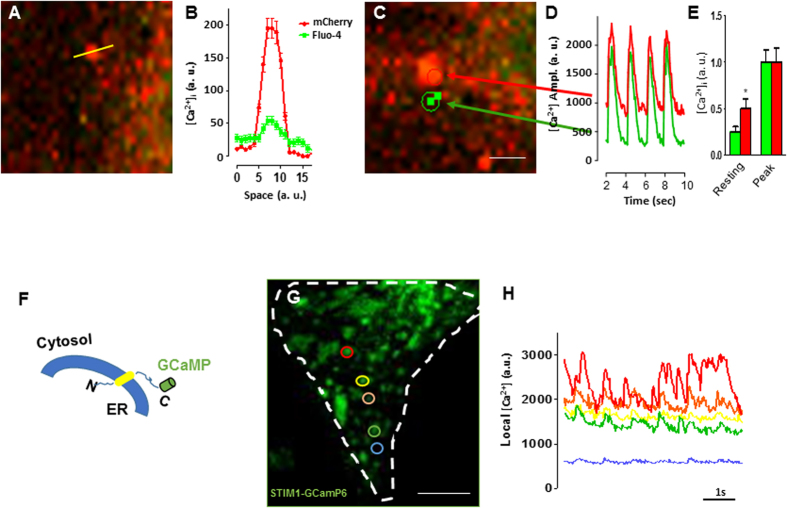
STIM1 clusters are associated with discrete Ca^2+^ microdomains. (**A**) Representative image from a live NVCM transfected with STIM1-mCherry, cells were loaded with fluo-4 at the day of the experiment. Laser line-scan confocal imaging was used to quantitate both mCherry and fluo-4 signals, a representative laser line-scan confocal is drawn on a single STIM1 cluster (yellow line). (**B**) Intensity variations along the laser-scan line of STIM1-mCherry domain (red trace) and the distribution of [Ca^2+^]_i_ (green trace) are plotted versus space. Traces show that [Ca^2+^]_i_ level is higher in correspondence with the center of STIM1 cluster. (**C**) Selected region of interest of subcellular STIM1-mCherry microdomain (red circle) and a region that does not contain STIM1 (green circle), the cell is also loaded with fluo4. (**D**) Traces of fluorescence changes during electrically evoked Ca^2+^ transient plotted over time. Traces represent intensity changes in ROI (from panel (c)), arrows and colors indicate the corresponding spots where the measurements were taken. Images were acquired with simultaneous excitation wavelengths of 488 nm and 587 nm. (**E**) Bar graph showing [Ca^2+^]_i_ transients peak analysis. [Ca^2+^]_i_ values were taken at the minimum between each peak “*resting*”; and at the maximum [Ca^2+^]_i_ concentration value at the peak of the transients “*Peak*”. (**F**) Cartoon representing the strategy followed to fuse GCaMP6 to the C-terminus of STIM1. (**G**) NVCM expressing STIM1-GCamp6, the white dashed line indicates cell edges, the color circle indicate selected STIM1-GCamp6 clusters. (**H**) STIM1-GCaMP6 fluorescent signals extracted from the correspondent subcellular clusters (panel (**G**)). The blue trace depict a spot where STIM1-GCamp6 is absent. Vertical scale values are given in arbitrary units (a.u.). Scale bar: panels (**A**,**C)** = 5 μm; panel g = 20 μm.

It has been reported that during cell migration STIM1 translocates to form clusters near the migration front, these clusters correlate with focal adhesions disassembling^18^,^26^. Intriguingly, it has been reported that local [Ca^2+^]_i_ increase is required to activate proteins that drive focal adhesion disassembly and cytoskeletal remodeling^31^,^32^. Therefore, we tested the hypothesis that STIM1 is responsible for myofilament degradation by increasing local Ca^2+^ concentration in proximity of the focal adhesions sites. Indeed, STIM1-mCherry formed clusters that were associated with discrete spots of higher fluo-4 generated signal (Fig. 6A). Line profile analysis of both STIM1-mCherry and fluo-4 fluorescence display a Gaussian distribution, with Ca^2+^ concentration decreasing by 68 ± 10% between the center and the periphery of the microdomain (Fig. 6B), this is suggestive of Ca^2+^ diffusing from the center of STIM1 cluster.

Further, dynamic fluorescence imaging of subcellular STIM1-mCherry microdomains in electrically paced fluo-4 loaded live-cell revealed a correlation between STIM1-associated local Ca^2+^ concentration and global Ca^2+^ transient (Fig. 6C). Interestingly, while a uniform rise of global Ca^2+^ transients was observed, in proximity of the STIM1 clusters cytosolic resting Ca^2+^ level lingered higher than the STIM1-free spots (Fig. 6D). These data suggest that STIM1-associated Ca^2+^ microdomains are responsible for the increase in global resting Ca^2+^ observed in NVCM overexpressing STIM1 (Fig. 6E).

Fluo-4 is largely used for measuring fast Ca^2+^ events at global and local level. However, the use of fluo-4 to measure spatially restricted Ca^2+^ signals is limited by dye diffusion. Therefore, estimation of spatial resolution of local changes in Ca^2+^ concentration at sites immediately near the STIM1 cluster is likely to be underestimated. To overcome this limitation, we fused the single-wavelength genetically encoded Ca^2+^indicator GCaMP6, which has spectral and kinetic characteristics similar to those of fluo4^33^, to the cytosolic C-terminal of STIM1 (Fig. 6F). The spatial distribution pattern of STIM1-GCaMP6 in NVCM was very similar to YFP-STIM1 or STIM1-mCherry pattern observed in NVCM (Fig. 6G), further confirming that STIM1 intracellular trafficking was not disrupted by the fusion with the Ca^2+^ biosensor. Spatially discrete fluorescent signals were detected in proximity of the STIM1-GCaMP6, time-lapse analysis of individual intracellular STIM1 microdomains showed spatially confined fluorescent signals that appeared to be different and distinct one from another (Fig. 6H). We conclude that STIM1-GCaMP6 confirms our previous observation that STIM1 cluster are associated with spatially segregated Ca^2+^ microdomains coexisting with, but independent from, the ECC.

### STIM1 deletion protects the heart from pressure overload-induced cardiac hypertrophy

**Figure 7:**
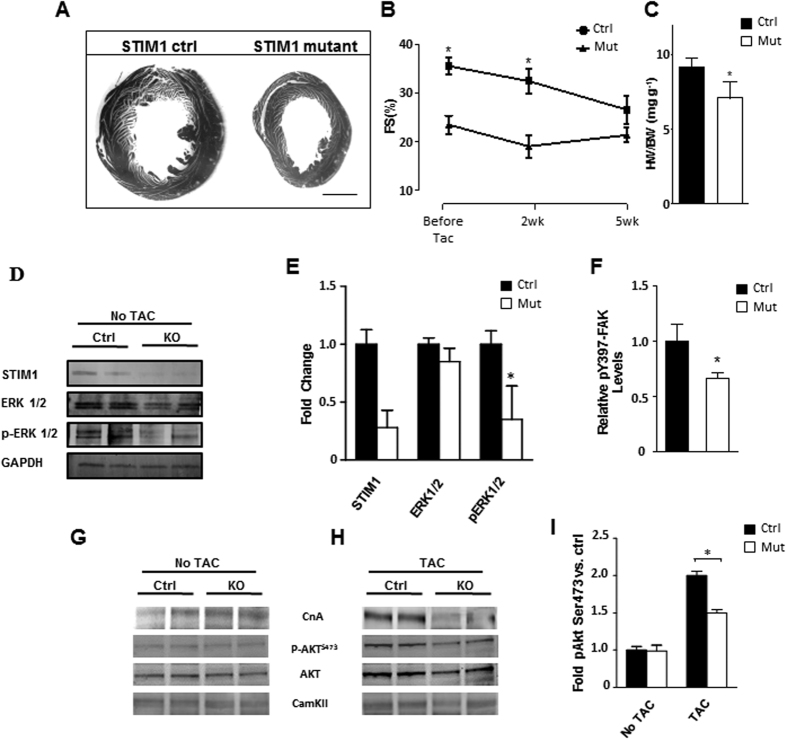
STIM1 mutant mice exhibit a blunted response to pressure-overload-induced cardiac hypertrophy. (**A**) Representative section of heart from STIM1 controls and STIM1 mutant. Left ventricular size was increased significantly in STIM1 control hearts. (**B**) Serial echocardiographic analysis and quantification of fractional shortening. (**C**) Postmortem ventricular weight/body weight ratio. Heart was harvested and ventricular weight is normalized for body weight. (**D**) Immunoblotting of isolated cardiomyocytes lysates blotted for STIM1, ERK1/2, activated ERK1/2 (pERK1/2) and GAPDH. (**E**) Quantitative analysis of four independent western blots, quantification was standardized relative to GAPDH signal, differences are reported in fold increase. (**F**) Summary of ELISA results from adult cardiomyocytes showing reduced p397-FAK in the STIM1 knockout cells, data were normalized for total FAK. (**G**) Representative immunoblotting of calcineurin (CnA), p-AKT (Ser473), AKT and total calmodulin dependent kinase II (CamKII) from heart lysates. (**H**) Same as in “panel **G**” but samples were extracted from mice after 14 days of TAC. (**I**) AKT densitometry results from multiple immunoblots, heart samples were harvested from mice after 14 days of TAC. Scale bar, 1.0 mm. Data are expressed as mean ± SEM, *P < 0.05.

The pathological implications of these findings were investigated in the setting where pathological cardiac hypertrophy would be an expected outcome. We used transverse aortic constriction (TAC) to impose chronic pressure overload and induce cardiac hypertrophy. Thus, after 5 weeks of transverse aortic constriction (TAC), control mice showed left ventricular hypertrophy as revealed by postmortem morphological analysis (Fig. 7A). Serial echocardiography further revealed gradual decline of cardiac function (Fig. 7B). In contrast, although STIM1 mutant mice exhibited impaired fractional shortening prior TAC, it showed preserved LV mass and cardiac function during the 5-week TAC period (Fig. 7B). Postmortem analysis of ventricular weight normalized for body weight were calculated to measure the extent of cardiac hypertrophy, STIM1 mutant mice displayed a ≈35% lower ratio than the control group (Fig. 7C). Pressure gradient across the aortic constriction was not different between STIM1 mutant and control banded mice. Hypertrophy was also attenuated in STIM1 KO cells as shown by cell length measurements (Supplemental Fig. 6A,B). After 5 weeks of TAC, analysis of vinculin in the “z-disk” revealed increased sarcomere distance in unloaded STIM1 KO cardiomyocytes. In contrast, control cells showed a higher number of sarcomere per space unit (Supplemental Fig. 6C–E). This suggest that while control cells have the ability to remodel their cytoskeleton by synthetizing new sarcomeres in response to chronic pressure overload, in STIM1 KO cells the mechanisms regulating cytoskeletal remodeling are impaired.

Cardiac remodeling during hypertrophy is accompanied by cytoskeletal binding and phosphorylation of FAK at the Tyr-397 residue with subsequent activation of the extracellular-regulated kinases1/2 (ERK1/2)^34^,^35^. Furthermore, ERK1/2 has been implicated in cardiac hypertrophy and it has been shown to be activated by SOCE^36^. Indeed western blot quantification revealed that ERK1/2 activity was markedly diminished in STIM1 mutant hearts, as indicated by reduced p-ERK1/2 levels (Fig. 7D,E). To evaluate whether FAK and the associated MAPK/ERK signaling are impaired in the STIM1 mutant mice, an ELISA-based assay was conducted to detect and quantify the level of both total FAK protein the phosphorylated FAK at tyrosine residue 397. We found that after 7 days of TAC, p397-FAK phosphorylation was reduced by ~24.26 ± 5% in cardiomyocytes isolated from STIM1 mutant mice when compared to the control counterpart (Fig. 7F).

Another signaling pathway affecting the growth response in cardiomyocytes, besides the Erks, is the Akt pathway which promotes cell survival and growth in response to extracellular signals^37^,^38^. Indeed, previous report showed that deficiency in FAK signaling abrogated the stretch-induced phosphorylation of both ERK and Akt, while upregulation of FAK was accompanied by activation of PI3K and Akt among others. Phosphorylation of Akt at serine 473 (Akt Ser473) is associated with maximal Akt activity, indeed, Akt Ser473 phosphorylation was significantly attenuated in STIM1 KO hearts subjected to pressure overload, when compared with the control counterpart (Fig. 7G–I). Of interest, calcineurin protein level is also increased in mice with TAC, but the increase is strongly attenuated in STIM1 KO hearts subjected to TAC. No differences were observed in total amount of CaMKII protein. Together, these data suggest that STIM1-mediated cardiac hypertrophy in response to TAC occurs through activation of FAK-Akt and ERK1/2 signaling pathways.

## Discussion

The precise role of STIM1 and the attendant Ca^2+^ signaling relevant to cardiac hypertrophy and ventricular function has largely been overlooked. Using a combination of *in-vitro* and *in-vivo* assays we have demonstrated that: *i*) STIM1 is necessary to maintain optimal cardiac function and plays a critical role in the development of pressure overload-induced cardiac hypertrophy; *ii*) at cellular level, STIM1 forms intracellular clusters responsible for the generation of spatially restricted Ca^2+^ microdomains required for cytoskeletal myofilament remodeling and activation of pro-hypertrophic signaling pathways. These results highlight STIM1-dependent Ca^2+^ microdomains involvement in myocyte growth, cytoskeletal remodeling and cellular signaling, whose mechanism of action coexists with [Ca^2+^]_i_ fluctuations involved in the excitation-contraction coupling.

The most striking effect on cardiac function resulting from STIM1 deletion was the LV dilatation associated with decreased cardiac contractility, which are consistent with those reported earlier in a constitutive STIM1 knockout mouse model^15^. We further revealed that STIM1 deletion caused reduced cell shortening, but had little effects on Ca^2+^ transients. Specifically, we observed an increased rate of cytosolic Ca^2+^ clearance in STIM1 KO cells which was associated with an increase of the NCX1protein level. A relationship between STIM1 and NCX1 has also been proposed by other groups^39^,^40^,^41^. While NCX1 increase can explain the faster intracellular Ca^2+^ decay, however, the nature of the association between STIM1 and NCX1 and the maintenance of the intracellular Ca^2+^ homeostasis in cardiomyocytes is unknown and further investigation is required in this matter.

A key piece of evidence that has emerged from our data is that cytoskeletal elements like vinculin and tubulin were profoundly altered in STIM1 KO cardiomyocytes. Indeed, these cytoarchitectural components play a significant role in the modulation of contraction speed and cytoskeletal mechanics^42^,^43^. In line with our observations, it has been reported that changes in tubulin density can affect cytoskeletal resistance by modifying the viscous component of the passive properties of cardiomyocytes contributing significantly to structural ventricular remodeling and dilatation^44^. These observation were amply corroborated by subsequent work on overexpressing systems. Indeed, STIM1-overexpression resulted in myofilament degradation and focal adhesion displacement. Myofilament disruption in proximity to STIM1 clusters was associated with a remarkable increase of non-myofibrillar space and impaired cell contractility, often resulting in subsequent cell detachment and death. STIM1 did not co-localize with vinculin and therefore we excluded a direct interaction between STIM1 and the focal adhesion complex. Further evidence linking STIM1 to the cytoskeleton and the focal adhesion turnover comes from cell migration studies, where STIM1 translocates in proximity of the focal adhesion to form clusters that disassemble the focal adhesion complexes allowing cells to migrate^18^,^26^.

Although cardiomyocytes have lost the ability to migrate, they still retain focal adhesion structures which become macromolecular scaffolds. These scaffolds functionally convert the mechanical forces into the biochemical signals that govern both cytoskeletal remodeling and activation of Ca^2+^ dependent signaling pathways involved in the hypertrophic gene transcription program^35^,^45^. Our data show that STIM1 deletion is associated with compromised cytoskeleton and FAK activation (pTyr397 FAK), this probably result in blockade of force transduction from integrin signaling to downstream pathways such as ERK1/2 and AKT, key players the genesis of cardiac hypertrophy^46^. Indeed, tamoxifen-induced STIM1 knockout mice were protected against pressure overload-induced cardiac hypertrophy. However, whether this model would be protected against the pathological increase in neurohumoral activation that is normally associated with vascular diseases remains to be confirmed.

NVCM overexpressing STIM1 provided valuable insight in understanding the relationship between STIM1, myofilaments distribution and sub-cellular Ca^2+^ signaling. In NVCM STIM1 formed clusters without the need of intracellular Ca^2+^ stores depletion. Similar findings were observed in adult cardiomyocytes. Pre-constituted “punctate” structures have been shown in skeletal muscle^47^, during myoblasts differentiation into myotubes^48^,^49^ and in adult cultured cardiomyocytes^13^,^39^. This is likely due to SR reorganization and reduced cytoplasmic space as muscle cells differentiate into a “contractile phenotype”. Furthermore, during normal Ca^2+^ transient we did not observe STIM1 rearrangements, which may be explained by the fact that during Ca^2+^-induced release not all Ca^2+^ from the sarcoplasmic reticulum is released into the cytoplasm. Hence, there may be enough stored Ca^2+^ at all times to prevent further STIM1 oligomerization, these results also suggest that STIM1 is not actively involved in the cardiac ECC.

STIM1 overexpression was associated with high cytoplasmic resting Ca^2+^ concentration. This result is confirmed by a recent published work using a transgenic mouse overexpressing STIM1, the authors reported an increase in cytoplasmic Ca^2+^ concentration in skeletal and cardiac muscles^14^. Indeed, our most surprising observation was that STIM1-clusters were associated with spatially restricted Ca^2+^ microdomains isolated from the global Ca^2+^ transients observed during the excitation-contraction coupling. Remarkably, STIM1-GCaMP6 allowed direct visualization of the Ca^2+^ “hot-spots” in proximity of the STIM1 clusters, revealing a very high correlation between Ca^2+^ concentration microdomains and STIM1 clusters. These findings are certainly intriguing and are further supported by studies showing that Ca^2+^ microdomains are required for selective activation of proteins that drive myofilament disassembly and cytoskeletal remodeling^31^. In this regard, it has been shown that calcium-activated calpains regulate proteolysis of focal adhesion kinases^50^ and that STIM1 overexpression in muscle correlates with significantly elevated calpains activity^51^. The present study raises the possibility that STIM1-dependent Ca^2+^ microdomain are instrumental for calpain activation and subsequent myofilament degradation.

Several laboratories have shown the presence of STIM1 and SOCE in neonatal and adult cardiomyocytes^3^,^10^,^11^,^52^. In line with these earlier reports, our data show evidence of STIM1-dependent Ca^2+^ entry that requires complete emptying of intracellular Ca^2+^ stores. However, whether SOCE is present in cardiomyocytes is still under intense debate. Zhao *et al.* reported absence of SOCE in adult cardiomyocytes^13^. Conversely, increased SOCE has been reported in cardiomyocytes overexpressing STIM1^14^. Nonetheless, the biophysical properties of the channels that are influenced by STIM1 in cardiomyocytes remain unknown. Furthermore, in contrast to our results, Zhao *et al.* did not observe STIM1 oligomerization upon Ca^2+^ store depletion (that is behind the pre-existing clusters). However, since Ca^2+^ dissociation from the STIM1 EF-hand motif is required in order for the cytoplasmic portion to rearrange and oligomerize^53^, it is not clear why upon depletion of Ca^2+^ from the stores STIM1 would not oligomerize. We attempted to induce STIM1 oligomerization by pharmacologically deplete SR Ca^2+^ store content, unfortunately, upon this treatment cells undergo to contractures introducing significant motion artifacts. To overcome this limitation we transfected the cells with the constitutive active STIM1-D76 mutant, which clearly show STIM1 oligomerization within the clusters.

Since STIM1 itself does not have channel properties, association with other molecular partners is crucial in order to generate Ca^2+^ microdomains. Although it is well established that Orai1 is the essential pore-forming unit of SOCC, an impressive number of proteins have been proposed to interact with STIM1: SERCA^54^,^55^,^56^, PMCA^57^, Ltype Ca^2+^ channel^58^,^59^, NCX^39^,^40^,^41^ and TRPC1^11^. Besides changes in NCX expression level other have reported that STIM1 overexpression negatively affect SERCA2a activity by inducing increased oxidative stress and altering mitochondrial bioenergetics^60^. This could explain why in our system STIM1 overexpression leads to increased cytosolic Ca^2+^ concentration. Moreover, STIM1 over-expression has been shown to attenuate PMCA-mediated Ca^2+^ clearance^57^, while STIM1 deletion enhances PMCA favoring a fast Ca^2+^ clearance rate. Thus, PMCA can play an active role on the maintenance of the calcium microdomains reported in cardiomyocyte.

Overall, we have characterized the Ca^2+^ signaling associated with STIM1 in both intact tissue and isolated cardiomyocytes. Our data suggest that STIM1 is required for maintaining optimal myocardial function. Further, we provide evidence that STIM1-dependent Ca^2+^ concentration microdomain are essential component of the focal adhesion complex that acts synergistically to trigger cytoskeletal remodeling and initiate the hypertrophic transcriptional program. In conclusion our data highlight a multicomponent signaling pathway that converge on the importance of Ca^2+^ microdomains required for downstream signaling activation and myofilament remodeling that are critically involved in the cardiac hypertrophic response.

## Materials and Methods

### Animal studies

All animal procedures described in this manuscript were reviewed and approved by the University of Tennessee Health Science Center Institutional Animal Care and Use Committee (IACUC). All methods were carried out in accordance with the approved guidelines.

### Transgenic Mice

Mice homozygous for STIM1-floxed alleles^4^,^24^ (abbreviated STIM1^flox/flox^) and transgenic α-MHC-MerCreMer mice (Jackson Laboratory, catalog number #5056) were crossed with STIM1^flox/flox^ transgenic mice for at least 7 generations to generate double-transgenic mice (α-MHC-MerCreMer-STIM1^flox/flox^). Intraperitoneal tamoxifen injections (40 mg/Kg/day) were given to sex and age matched mutant (α-MHC-MerCreMer-STIM1^flox/flox^) and control mice (α-MHC-MerCreMer-STIM^wt/wt^). Mice were injected 3 to 4 weeks after birth, at least 28 days of rest post-injection were allowed before mice were used for experiments.

### Immunofluorescence staining and confocal microscopy analysis

Neonatal rat cardiomyocytes were isolated from 1–3 days old rat pups and were grown on fibronectin-coated coverslips. Cells were infected with the indicated lentivirus or transfected using lipofectamine 3000 (Life technologies). For immunostaining cells were washed once with ice-cold PBS and fixed in 3.7% with buffered formaldehyde for 10 min. After three washes with PBS, cells were treated for 10 min with 0.2% Triton X-100 in PBS and incubated 30 min with PBS/3% BSA. Cells were washed three times with PBS then incubated with primary antibodies as follows: 1 h with either anti-vinculin (Cell Signaling, 1/100) anti-β-tubulin (Abcam, 1/100) in PBS, 0.2% BSA. Rhodamine phalloidin (1:200) was used to show actin distribution, while GFP-conjugated anti-GFP antibody (Thermo Scientific) was used against YFP-STIM1. Secondary antibodies were chosen accordingly, cells were incubated with a TRITC-conjugated anti-rabbit secondary antibody (Thermo Scientific), with 5 U/ml of Alexa 488. After a 2-h incubation, excess of secondary antibodies were washed out and coverslips were mounted onto glass slides with anti-fade mounting medium. Confocal microscopy was performed with a Zeiss LSM 510 Pascal or Zeiss LSM710 confocal microscopes. Image-acquisition settings (laser intensity, pinhole size, and amplifier gain) were strictly maintained constant for all images taken on the same day to allow quantitative, paired comparisons.

### Data Analysis

Images from immunoblotting and fluorescence intensity, were evaluated by scanning and measuring the density of the bands (ImageJ 1.48v, Wayne Rasband, National Institute of Health, USA). The data were expressed as means ± SEM unless otherwise indicated. The significance of comparison of mean values was determined by ANOVA or unpaired Student *t* test where appropriate (Graphpad, Prism5), using a significance level of *P* < 0.05 and *P* < 0.01.

### Other methods

For additional methods and details see supplemental material.

## Additional Information

**How to cite this article**: Parks, C. *et al.* STIM1-dependent Ca^2+^ microdomains are required for myofilament remodeling and signaling in the heart. *Sci. Rep.* **6**, 25372; doi: 10.1038/srep25372 (2016).

## Supplementary Material

Supplementary Information
